# Excellent UV-Light Triggered Photocatalytic Performance of ZnO.SiO_2_ Nanocomposite for Water Pollutant Compound Methyl Orange Dye

**DOI:** 10.3390/nano11102548

**Published:** 2021-09-28

**Authors:** Sunil Rohilla, Ankita Gupta, Vibhor Kumar, Suman Kumari, Michal Petru, Nesrine Amor, Muhammad Tayyab Noman, Jasvir Dalal

**Affiliations:** 1 Department of Physics, Chaudhary Ranbir Singh University, Jind 126102, India; sunilrohilla@crsu.ac.in (S.R.); sumankkharb@gmail.com (S.K.); 2 Department of Physics, Adarsh Mahila Mahavidyalaya, Bhiwani 127021, India; ankita13994@gmail.com; 3 Department of Electrical Engineering, Northern Illinois University, Dekalb, IL 60115, USA; vibhorsoni123@gmail.com; 4 Department of Machinery Construction, Institute for Nanomaterials, Advanced Technologies and Innovation, Technical University of Liberec, Studentská 1402/2, 46117 Liberec, Czech Republic; michal.petru@tul.cz (M.P.); nesrine.amor@tul.cz (N.A.)

**Keywords:** water pollutant, Rietveld refinement, Tauc’s plot, photocatalytic activity, methyl orange

## Abstract

The photocatalytic activity of eco-friendly zinc oxide doped silica nanocomposites, synthesized via a co-precipitation method followed by heat-treatment at 300, 600, and 900 °C is investigated. The samples have been characterized by employing X-ray diffraction method, and further analyzed using the Rietveld Refinement method. The samples show a space group P63mc with hexagonal structure. The prepared composites are tested for their photocatalytic activities for the degradation of methyl orange-based water pollutants under ultra-violet (UV) irradiation using a 125 W mercury lamp. A systematic analysis of parameters such as the irradiation time, pH value, annealing temperatures, and the concentration of sodium hydroxide impacting the degradation of the methyl orange (MO) is carried out using UV-visible spectroscopy. The ZnO.SiO_2_ nanocomposite annealed at 300 °C at a pH value of seven shows a maximum photo-degradation ability (~98.1%) towards methyl orange, while the photo-degradation ability of ZnO.SiO_2_ nanocomposites decreases with annealing temperature (i.e., for 600 and 900 °C) due to the aspect ratio. Moreover, it is seen that with increment in the concentration of the NaOH (i.e., from 1 to 3 g), the photo-degradation of the dye component is enhanced from 20.9 to 53.8%, whereas a reverse trend of degradation ability is observed for higher concentrations.

## 1. Introduction

To meet the demand and supply of developing countries, numerous small and large-scale industries are flourishing. The fast-growing industries produce a large number of toxic chemical wastes due to a lack of proper management and knowledge. The chemical wastes contain several non-biodegradable elements and other water pollutants, which are harmful to both aquatic and human life. Major water pollutants involve dye molecules used in pharmaceuticals, paper, leather, plastic, cosmetics, food, and textile industries [1,2]. It is reported that the yearly generation of the synthetic dye is greater than 7 × 10^5^ tons, out of which around 2 × 10^3^ tons of dyes are discharged in water bodies only as textile waste [3]. Among many dyes, azo dyes like methyl orange (MO) are used in laboratories as pH indicators, water-soluble dye products in textiles, printing, paper, pharmaceuticals, and food industries [4]. MO displays an orange colour in the basic medium and a red colour in the acidic medium [5]. Due to its high solubility, it is difficult to remove MO from solvents using a conventional treatment system [6]. Drinking MO contaminated water may cause vomiting, shock, irregular heart rate, jaundice, and tissue necrosis in humans [7]. Thus, it is important to remove the residual MO content from wastewater produced by industries. Numerous water treatment techniques, such as chemical oxidation, adsorption, precipitation, coagulation, electrolysis, photo-degradation, and many more, are employed for the filtration and purification at the industry level before releasing it into the environment [8,9,10].

In recent times, metal oxides are reported as good photocatalysts due to their excellent photocatalytic activity, high stability in a watery environment, and low cost [11,12], and are the most widely used for wastewater treatment [13,14,15,16]. Lu et al. have reported on the TiO_2_/biochar composite for photocatalytic degradation of MO, and have illustrated the good catalytic performance of TiO_2_ nanocomposites [17]. Molkenova et al. examined the photocatalytic performance of hollow CuO microspheres for the degradation of pollutant Rhodamine B dye [18]. With ZnO having remarkable physical, chemical, and optical properties, as well as an easy scale-up and complete mineralization capacity, it is purposefully used in the catalytic processes for the elimination of organic-dye pollutants [19]. Wang et al. used a porous graphene/ZnO nanocomposite to decolor MO and confirmed enhanced efficiency as compared to bare ZnO nanoparticles [20]. Ali et al. reported the photo-degradation of the MO using ZnO/SnO_2_ nanocomposites. Their studies revealed a decrement in the photo-degradation efficiency of ZnO/SnO_2_ nanocomposites with an increase in the annealing temperature [21]. Albiss et al. fabricated ZnO nanorods over the activated carbon fiber composites and studied the photocatalytic performance [22]. It is reported that the photocatalytic activities depend on surface area, absorption of UV-light, and charge carrier recombination, and the photocatalytic efficiency can be enhanced by improving these properties.

However, while ZnO is widely used for photocatalytic activities, it has some basic problems such as a high optical band gap (3.37 eV) and a high recombination rate of electron-hole pairs (EHPs), which diminish its photocatalytic efficiency [23,24]. Therefore, it is always preferable to mix doped ZnO with some porous matrix or system, such as silica dioxide (SiO_2_). The doping of SiO_2_ leads to high mass transport, which causes higher selectivity and adsorption of the pollutants [25]. Additionally, the core of the silicon dioxide will act as an electron-trapping centre. These trapping centres deal with the electron generated from ZnO due to the process of photon irradiation and excitation, which reduces the recombination rate of EHPs, and in turn, contributes to an enhanced photocatalytic performance [26].

In light of the above, the present work reports on the synthesis of ZnO.SiO_2_ nanocomposites, the examination of their structure, and photo-degradation of methyl orange pollutant of the nanocomposite. This report explains the correlation between the structure and the parameters such as temperature, pH, irradiation time, and NaOH concentration affecting the photo-degradation performance of nanocomposites.

## 2. Experimental Details

### 2.1. Materials Used

For the synthesis of this photocatalyst material, zinc nitrate hexahydrate (Zn(NO_3_)_2_·6H_2_O, ethanol (C_2_H_5_OH), silicon oxide (SiO_2_), sodium hydroxide (NaOH), HCl, and methyl orange (C_14_H_14_N_3_NaO_3_S) were procured from the Merck, Bengaluru, India. All chemicals were of analytic grade and were used as procured.

### 2.2. Synthesis

**Scheme 1:**
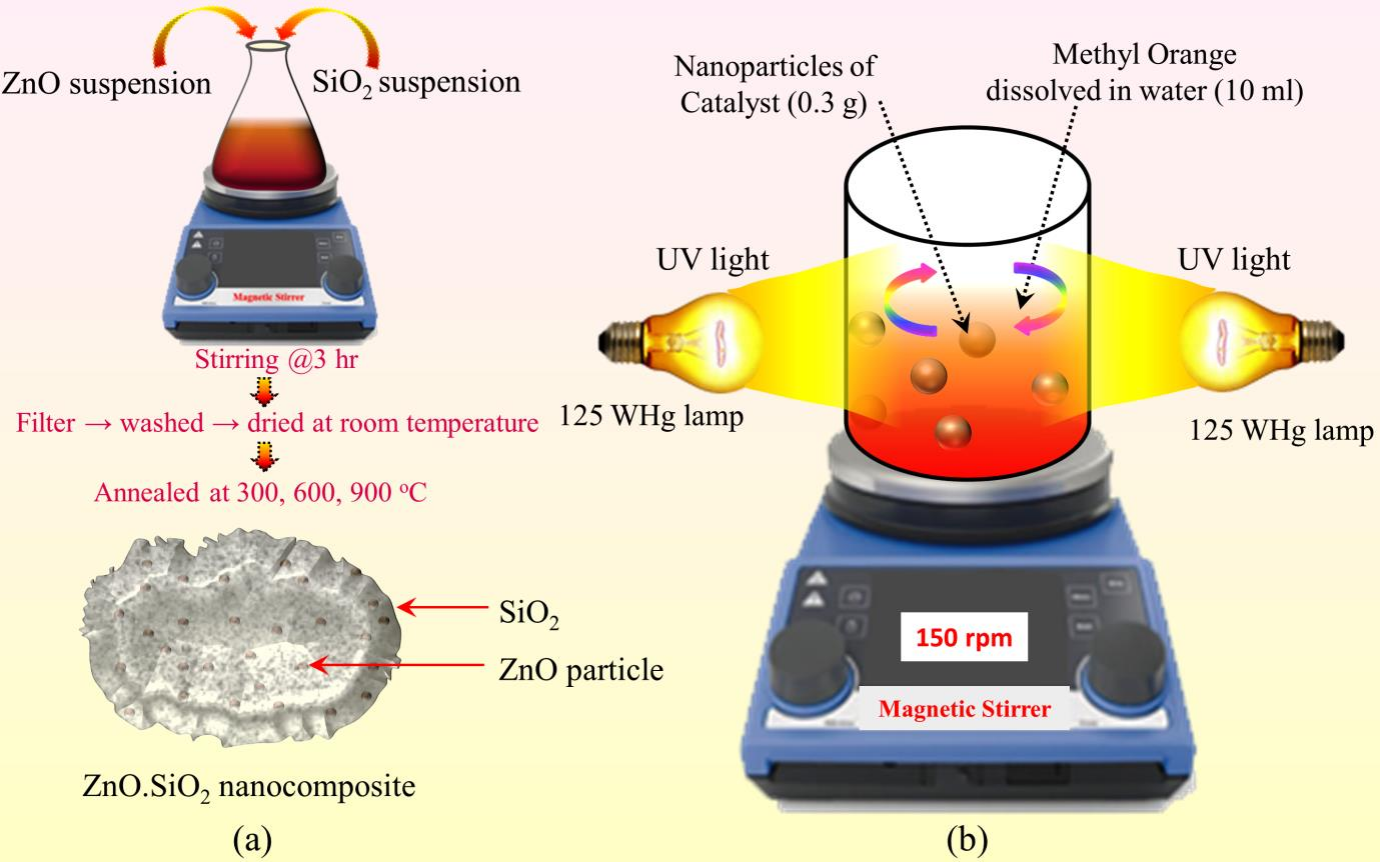
Schematic diagram of (**a**) preparation of SiO_2_.ZnO nanoparticles with the expected morphology (ZnO nanoparticles embedded into SiO_2_ porous structure) and, (**b**) photocatalytic activity set-up.

The synthesis of the ZnO.SiO_2_ nanocomposite involves three steps. The first step involves the preparation of ZnO nano-suspension. For this, 0.1 M NaOH solution was prepared in 50 mL distilled water and was added to 0.1 M of Zn(NO_3_)_2_·6H_2_O dissolved in 100 mL ethanol, following the procedure reported in our previous work [27]. Secondly, the preparation of SiO_2_ nano-suspension was prepared by mixing 0.3 M SiO_2_ in 50 mL 0.6 M NaOH, which resulted in Na_2_SiO_3_. The prepared solution was drop-wise added (using the piezoelectric nozzle of 0.05 mm and drop rate of 0.02 mL/s) to 50 mL 0.6 M HCl, which resulted in white precipitates of SiO_2_. The obtained suspension was stirred at 8000 rpm for 3 h at 50 °C. Afterward, NaCl was removed from the suspension by washing it several times using distilled water [28]. For the synthesis of the ZnO.SiO_2_ nanocomposite, both suspensions were mixed and stirred [28]. The white precipitates were filtered with grade 5 Whatman filter paper and then washed and dried in air. By following the procedure (Scheme 1a) two samples (0.1 M)ZnO.(0.3M)SiO_2_ (ZS1) and (0.05 M)ZnO.(0.3 M)SiO_2_ (ZS2) were prepared; furthermore, these samples were annealed at the different temperatures of 300, 600, and 900 °C for 2 h. For ease of understanding, the samples annealed at 300, 600, and 900 °C are named as ZS1_300_, ZS1_600_, and ZS1_900_, respectively.

### 2.3. Characterization

For the determination of the crystallite size and lattice constant, the X-ray diffraction (XRD) patterns of the samples were acquired with an X-ray diffractometer (Philips PW/1710), operated at 50 kV and 40 mA in range of 10 to 70 degrees. The XRD data was analyzed through Rietveld refinement for a perfect matching of phases and to determine various lattice parameters, dislocation density, microstrain, Wyckoff positions, bond length, and bond angle, etc. For morphological analysis, transmission electron microscopy was performed on a Hitachi (H-7500) instrument. Fourier transform infrared spectrometer (Bruker compact alpha-II) was used for recording the spectra of nanocomposites in a range of 4000–400 cm^−1^ using Kbr pallets. The UV-Vis absorption spectra of the prepared samples were acquired with a Lambda 750 spectrophotometer (Perkin) in a wavelength from 300 to 700 nm. The surface area (S_BET_) was evaluated using N_2_-adsorption measurements performed on a Quantachrome Instruments at 77.3500 K. Nitrogen and carbon were used as adsorbate and adsorbent, respectively.

### 2.4. Photocatalytic Activity

The photocatalytic properties of ZnO.SiO_2_ nanocomposite were evaluated by testing the photo-degradation of methyl orange (MO) in water using UV irradiation (125 WHg lamp). In a typical reaction, 30 mg ZS1 composite and 10 mL of 30 mg/80 mL of methyl orange dye aqueous solution were stirred using a magnetic stirrer for an hour to establish the adsorption/desorption equilibrium of MO on the surface of catalyst before the irradiated process. For this experiment, two mercury lamps were placed 5 cm away from the magnetic stirrer on which the solution of catalyst, as well as dye, were stirred, as shown in Scheme 1b. The effect of pH (at 7 and 12) on photocatalytic activity was analyzed. The pH of methyl orange dye solution (MODS) was controlled by adding diluted Nitric acid and NaOH. For this analysis, solutions of ZS1 (ZS1_300_, ZS1_600_ and ZS1_900_) and MODS were prepared with pH values of 7 and 12. At consequent intervals of time (i.e., 0 min, 20 min, 40 min, 60 min, 100 min) 2 mL of samples were extracted from each solution and analyzed using UV–Vis spectroscopy. The effect of NaOH conc. on the photo-degradation of dye was also evaluated. For this analysis, solutions of ZS1_300_ and MODS were prepared with different conc. of NaOH (1 g, 2 g, 3 g, and 4 g). Afterward, the slurry was not stirred, and the resulting dye content was determined at the end of the experiment. The photo-degradation percentage of methyl orange is calculated using Equation (1) [29]

(1) $$\mathrm{Photo}-\mathrm{degradation}\;\left(\mathrm{removal}\;\mathrm{efficiency}\right)=\frac{A_{o}-A_{t}}{A_{o}}\times 100$$

where, A*_o_* is the initial concentration of MO and A*_t_* is the concentration of MO after time *t*. The experiment has been repeated again to ensure the reproducibility of the results.

### 2.5. Zero-Point Charge

**Figure 1:**
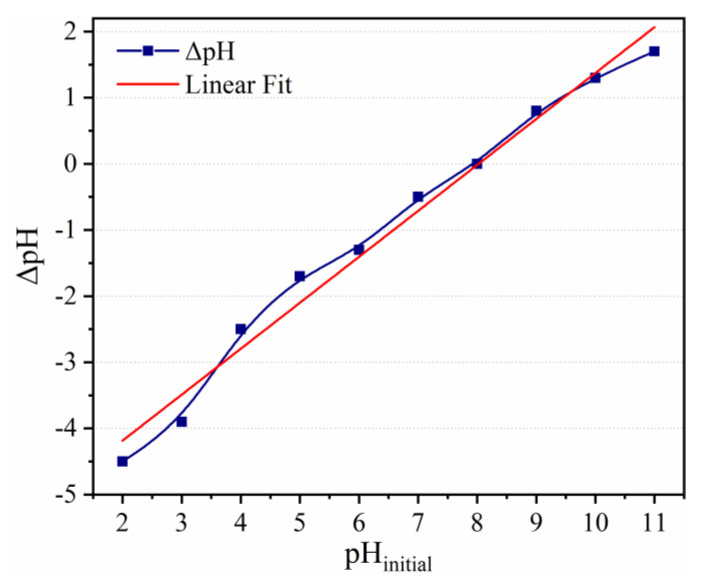
Plot of ΔpH versus pH_initial_ for ZS1_300_.

The value of zero-point charge (ZPC) was calculated with the help of salt titration method. The ZPC value of the catalyst was determined using a 10 mol solution of NaClO_4_. In this method, 0.2 g of catalyst was taken in 10 mL deionized water, and 0.01 mol/L NaOH was added to get the required pH. The suspension was taken in ten different beakers and the pH of the suspensions was initially set to different values from two to 11. This pH value is denoted as pH_initial_. The sample of each beaker was stirred briskly for 24 h at room temperature, and the value of pH was recorded. This pH value is denoted as pH_final_. The ZPC is obtained from the plot, between ΔpH (pH_final_–pH_initial_) and pH_initial_ as depicted in Figure 1. For ZPC, ΔpH needs to be zero. The ZPC of ZS1_300_ is investigated and its value is found as 8 [30].

The value of ZPC implies that surface of the catalyst is positively charged at pH < pH_ZPC_, and for pH > pH_ZPC_ will be negatively charged. Therefore, ZPC is considered an important feature for photocatalytic activity as it affects the surface property of catalyst. Under basic pH conditions, the negatively charged pollutant compounds will be repelled from the negatively charged catalyst surface, which results in to decrease in absorption of the pollutant compound. As a result, the degradation efficiency of the sample falls at a higher pH [31].

## 3. Results and Discussion

### 3.1. XRD Analysis

**Figure 2:**
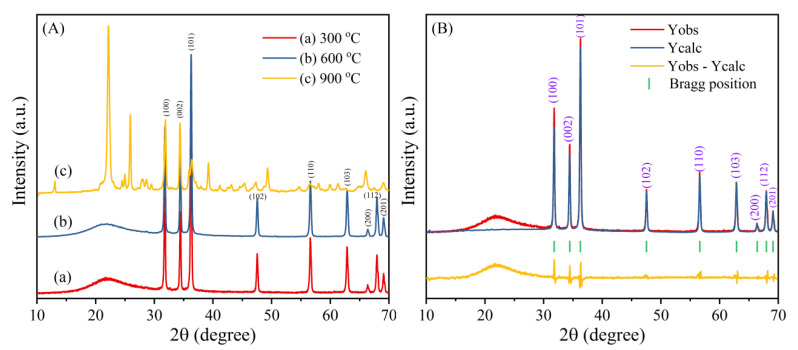
(**A**) XRD patterns of ZS1 annealed at various temperatures and (**B**) Rietveld Refined XRD pattern of ZS1_300_ nanocomposite.

The XRD patterns of ZS1_300_, ZS1_600_ and ZS1_900_ samples are shown in Figure 2A. It can be observed from Figure 2A(a,b) that the XRD patterns of both samples contain various well-defined reflections at 2θ at 31.68°, 34.32°, 36.26°, 47.56°, 56.59°, 62.87°, 66.44°, 67.95°, and 69.08° correspond to Miller planes (100), (002), (101), (102), (110), (103), (200), (112), and (201) respectively, confirming the formation of ZnO [32,33,34]. In both diffraction patterns, a broad hump is present at 2θ~21.98°, which reveals the presence of amorphous silica. The observed peaks in in the diffraction pattern reveal the formation of the hexagonal structure of ZnO embedded in silica matrix, with the space group P63mc (JCPDS card no. 790205).

However, the XRD pattern of the sample ZS1_900_ also contained some new peaks (Figure 2A(c)). The peak centred at 2θ (reflection planes) at ~22.06° (101), 28.76° (111), 31.70° (102), 36.14° (200), 45.44° (202), 47.28° (113), 48.72° (212), 62.18° (302), 65.02° (312), and 66° (204), which may be ascribed to Tetragonal Cristobalite (JCPDS File No. 82-1408). In the same pattern, some of the peaks cantered at 2θ (reflection planes) at ~31.70° (113), 34° (410), and 38.80° (223) may be ascribed to Hexagonal Zinc silicate (Zn_2_SiO_4_) (JCPDS File No.37-1485).

The value of structural parameters like plane (hkl), crystallite size (*D*), d-spacing, full width at half maximum (*β*), intensity, microstrain (*ε*) and the dislocation density (δ) of ZnO.SiO_2_ composite are obtained from XRD analysis and are shown in Table S1. The crystallite size (*D*) is calculated by using Scherrer’s formula as:

(2) D=(0.9×λ)/( β×cosθ)

The average crystallite size is calculated from the three peaks of highest intensity in the pattern, which was characterized by lattice plane (100), (002) and (101) at 2*θ* ~ 31.85, 34.55 and 36.35°, respectively inserted in Table S1. Dislocation density (*δ*) is calculated as [35]:

(3) $$\delta =1/D^{2}$$

and microstrain (*ε*) value of the nanocomposite ZnO.SiO_2_ is evaluated as,

(4) ε=βcos θ/4

The Rietveld refined X-ray diffractogram of ZS1_300_ nanocomposite is shown in Figure 2B. For the quantitative analysis of the phase present in the sample, Rietveld refinement was carried out of the XRD data. The atomic position and isothermal parameters for Zinc and Oxygen are fixed and the other parameters like lattice constants, shape parameters, and scale factors are considered as free parameters during the time of fitting. The parameters, such as background factors and scale factors, are refined in the first step of the Rietveld refinement. The background is fitted using a linear interpolation method and pseudo-voigt is chosen for peak profile. In the succeeding stage, the structural parameters (lattice parameters, width parameter, preferred orientation, asymmetry, and atomic coordinates, etc.) are refined.

Figure 2B approves the hexagonal structure of ZnO embedded in SiO_2_. The Wyckoff positions parameters $\left(\frac{x}{a},\frac{y}{b},\frac{z}{c}\right)$ of atoms obtained from refinement are tabulated in Table S2. The norm’s factor for the best fit, such as the Reliability factors (R factors), Profile R factor (R_B_ = 13.6), Weighted R Factor (R_wp_ = 14.3), Expected values (R_exp_ = 7.17), Bragg R Factor (R_B_ = 6.02), R_f_–Factor (R_B_ = 2.78) and Goodness fit factor (χ^2^ = 3.9) value obtained from Rietveld refinement, confirm the precision of the Rietveld refinement analysis. The value of lattice parameters obtained from the refinement are as follows: a=b=3.249 (Å); c=5.2058 (Å); α = 90°; $\gamma =120^{o}$, and ratio of c/a is 1.6022. The bond lengths between different atoms and bond angles between them are evaluated and presented in Table S3.

The type of the grain boundaries may play an important role in the photocatalytic mechanism. The grain boundary specific area (*S_a_*) i.e., the ratio of the area enclosed by the grain boundary and volume of grain, is estimated as [36]

(5) $$S_{a}=\frac{1.65}{D}$$

where, *D* is the crystallite size estimated from XRD studies using Equation (2). The Equation (5) is valid for both single and polycrystalline forms of ZnO system having a random distribution of shapes and grains. The values of *S_a_* are tabulated in Table 1. It is observed that the grain boundary-specific area is decreased with an increase in temperature. The higher value i.e., 4.445 × 10^7^ m^2^ m^−3^ of *S_a_* is calculated for the ZS1 sample annealed at 300 °C.

With a change in the precursor’s concentration i.e., sample ZS2, it is observed from the XRD patterns of ZS2_300_ and ZS2_600_ for the ZnO phase that the crystallite size decreased compared to ZS1_300_ and ZS1_600_, respectively. The XRD data for ZS2_300_ and ZS2_600_ are provided in the Supplementary File. The diffractogram of ZS2_900_ was found to have marginal changes compare to ZS1_900_. The phase structure observed for ZS2 samples is the same as observed in the case of ZS1 samples.

### 3.2. TEM Analysis

**Figure 3:**
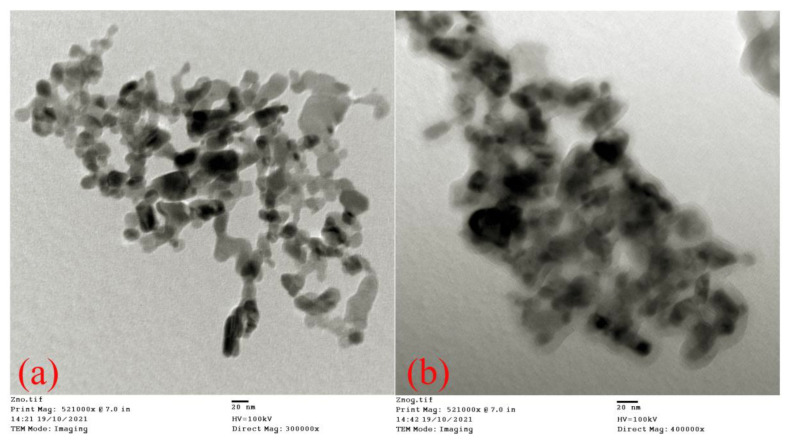
Transmission electron microscopy images of ZS1_300_ (**a**) lower magnification and (**b**) higher magnification.

Transmission electron microscopy (TEM) is a utile technique for the analysis of particle size and the morphology of materials. The microscopic images of ZnO.SiO_2_ nanocomposites annealed at 300 °C are depicted in Figure 3. The average particles size is found to vary between 20 to 30 nm, which is nearly analogous to the crystallite size calculated from the XRD results. From the figure, a uniform morphology has been observed in which the particles are appearing roughly as spherical shapes. The micrograph shows the weak aggregation of ZnO particles that confirm the presence of particles in the silica matrix. Some particles are observed to possess sharp grain boundaries and a clear morphology in the silica matrix.

### 3.3. FTIR Analysis

For the further investigation of the molecular structures of ZS1_300_, ZS1_600_ and ZS1_900_ nanocomposites, Fourier-transform infrared (FTIR) spectroscopy studies are carried out and the FTIR spectra of these samples are shown in Figure 3.

**Figure 4:**
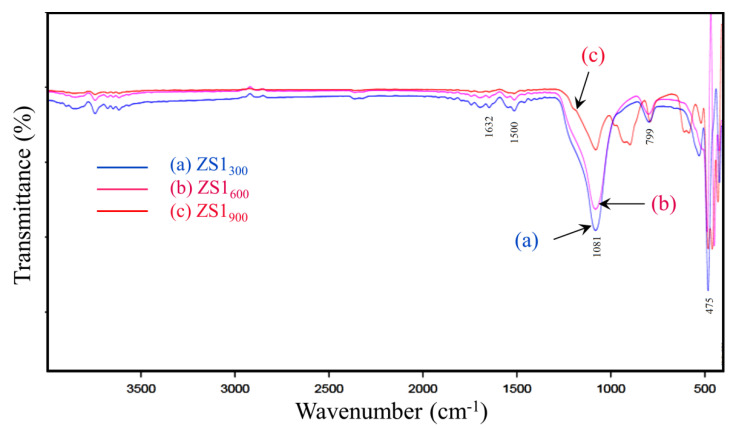
Fourier transforms infrared spectra of (**a**) ZS1_300_, (**b**) ZS1_600_, and (**c**) ZS1_900_ nanocomposite.

Characteristic peaks of these samples are observed at 1081 and 799 cm^−1^, which represent the stretching vibration mode and symmetric stretching of the Si-O-Si group, respectively [26]. The peak at around 475 cm^−1^ in the spectra is due to the vibration of the Zn, and the O bond confirms the presence of ZnO in the samples [37]. The presence of bonds at 1500 cm^−1^ and 1632 cm^−1^ are attributed to the stretching vibration of the (−OH) group attached with Si and ZnO, respectively [38]. The hydroxyl group results from the hygroscopic nature of ZnO and Si that come during the synthesis. It is also observed that the content of hydroxyl group in the sample decreased with an increase in temperature, suggesting the presence of impurities mostly near the surfaces of ZnO and Si. Moreover, it can be found that the peak at 1081.98 of ZS1_300_ sample had a strong intensity. However, for ZS1_600_ and ZS1_900_, the peak intensity decreased due to the decomposition of hydroxyl group Figure 4a,b. In the spectrum of ZS1_900_ sample (Figure 4c), some new peaks are also observed, which confirm the formation of some intermediate phases of ZnO and cristobalite (crystallite form of silica). The observed changes in the FTIR spectra of the nanocomposites samples are well-matched with the findings of XRD analysis.

### 3.4. UV-Visible Analysis

**Figure 5:**
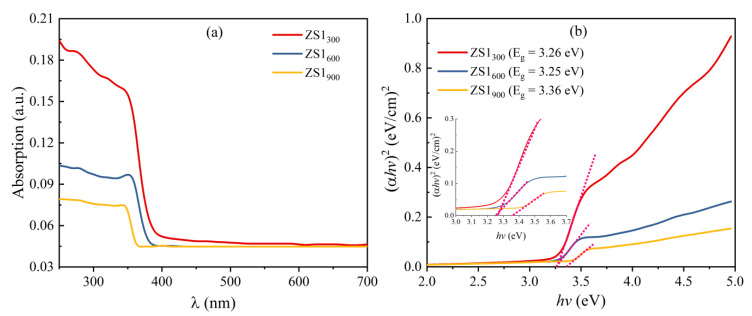
(**a**) UV plots (**b**) Tauc’s plot of ZS1_300_, ZS1_600_ and ZS1_900_.

For the examination of the optical band gap of the ZS1_300_, ZS1_600_ and ZS1_900_ samples, UV-Visible spectroscopy is performed, as shown in Figure 5. It is seen that band edge shifted to a lower wavelength edge (i.e., blue shift), suggesting an alteration in the optical band gap. Thereby, to estimate the energy band gap (*E_g_*) of the catalyst samples, Tauc’s relation the spectra (250–700 nm) is used, which is given by

(6) $${\left(\alpha h\nu \right)}^{1/n}=A\left(h\nu -E_{g}\right)$$

where α, h, n and A are the absorption coefficients, Planks constant, power factor for the transition mode, and constant, respectively. For the allowed direct transitions, n is taken as 1/2. The band gap values for the nanocomposites samples are determined by plotting ${\left(\alpha h\nu \right)}^{1/n}$ versus hν, as shown in Figure 5.

The estimated values of *E_g_* for the ZS1_300_, ZS1_600_, and ZS1_900_ nanocomposites are 3.26 eV, 3.25 eV, and 3.36 eV, respectively. The optical band gap of ZS1_300_ is found to be smaller than the characteristic value of ZnO, i.e., 3.37 eV at room temperature. This may be due to the occurrence of intrinsic defects like oxygen vacancies and zinc interstitials in the ZnO [36,39]. The ZS1_900_ sample has a higher optical band gap (very near to 3.37 eV) which attributes to an increase in crystallite size, as can be seen from XRD studies. The observed values of *E_g_* w.r.t. crystallite size are tabulated in Table 1.

### 3.5. BET Analysis

**Figure 6:**
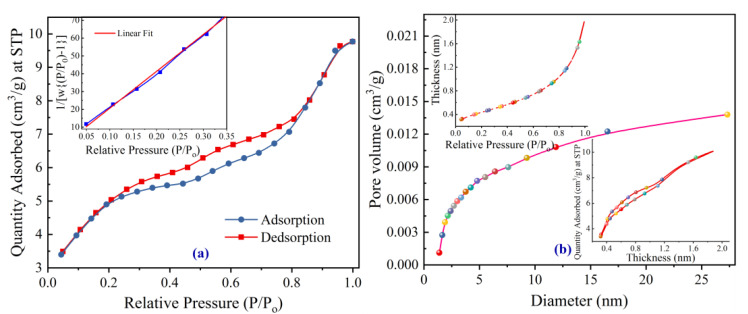
BET isotherm of ZS1_300_ (**a**) adsorption and desorption curve and (**b**) pore size distribution, the thickness of deposited layer (inset) & adsorption quantity versus size of the pore (inset).

Surface area is an important parameter that affects photocatalytic activity due to the surface dependency of the process. With a larger surface area, the active sites are larger and with better photocatalytic activities. Therefore, to measure the surface and porosity of the ZS1_300_ and ZS1_900_ nanocomposite samples, the Brunauer-Emmett-Teller (BET) measurements are performed in a nitrogen environment. The nitrogen (N_2_) adsorption-desorption isotherm curves of the ZS1_300_ sample are shown in Figure 6a. The shape of adsorption-desorption curves depicts the features of isotherm of type IV (as per IUPAC classification) with H4 hysteresis, which reflects the mesoporous kind nature of the samples under discussion. The BET curve having three main segments, i.e., (i) concave-shaped for relative pressures from 0.05 to 0.1, (ii) linear-shaped up to 0.3, and (iii) convex-shaped for above 0.3, the (ii) or linear-shaped segment, as shown in inset of Figure 6a, denotes a realization of the first monolayer of the gas molecules and commencement of the second layer. The linear segment of the BET curve is known as the ‘B’ point, which is further used to calculate the specific surface area. From the BET equation:

(7) $$\frac{p/p^{o}}{w\left(1-p/p^{o}\right)}=\frac{1}{w_{m}C}+\frac{C-1}{w_{m}C}\left(p/p^{o}\right)$$

whereas, w denotes the volume of adsorbed gas at normal temperature (T) and pressure (P); $w_{m}$ represents the volume of adsorbed gas for monolayer per gram of adsorbent; P° denotes saturated vapor pressure of N_2_ at normal temperature; *C* refers to a constant depending on the *H_a_* (heat absorbed by the monolayer of N_2_); and *H_L_* (liquefaction parameter of the gas) in the range of 0.05–0.3 of relative pressure, given by flowing relation

(8) $$C=exp\left(\frac{H_{a}-H_{L}}{RT}\right)=exp\left(\frac{ƊH}{RT}\right)$$

The Equation (7) represents an equation of a straight line in between $\frac{p/P_{0}}{w\left(1\;–P/P_{0}\right)}$ vs. $\frac{P}{P_{0}}$ with slope $\;\left(\frac{C-1}{w_{m}C}\right)$ and intercept of $\;\left(\frac{1}{w_{m}C}\right)$ having the values of 206.87578 g/cm^3^ and 0.0088 g/cm^3^, respectively. The volume of the gas molecules monolayer ($w_{m}$) can be obtained using the values of slope and intercept of the curve in the straight-line portion of the isotherm. The specific surface area can be calculated using the relation, $S_{\mathrm{BET}}=w_{m}A_{m}N$, where N is the Avogadro number and *A_m_* is the molecular cross-sectional area (for nitrogen; *A_m_* = 0.162 nm^2^). The measured values of the specific surface areas (S_BET_) for the samples ZS1_300_ & ZS1_900_ are found to be 16.8 m^2^/g and 0.001 m^2^/g, respectively. The decrease in specific surface area with annealing temperature can be attributed to the penetration of ZnO nanocrystallites into the porous structure of SiO_2_. The filling of the closed pores of SiO_2_ with ZnO resulted in the decrease in porosity of the nanocomposite samples. This can easily be predicted and corroborated with the change in XRD pattern from ZS1_300_ to ZS1_900_, wherein the XRD pattern of ZS1_300_ is vastly matched with the pure ZnO hexagonal structure. On the flip side, the XRD pattern of ZS1_900_ consists of the sharps reflections of ZnO along with the well-visualized reflection of SiO_2_, which points to the mixed phase of the nanocomposite.

The respective pore size distribution curve for the sample under discussion is obtained with the adsorption particulars employing the Barrett-Joyner-Halenda (BJH) method and depicted in Figure 6b. This figure confirms the mesoporous nature of the prepared composite. The insets depict the change in quantity adsorbed as a function of the deposited layer thickness and the change in layer thickness with relative pressure. It can be seen that the composite contains a total pore volume of 0.01667 cm^3^/g and an average pore diameter of 3.838 nm. The ZS1_300_ nanocomposite shows a higher specific surface area and it is reported that a higher surface area is advantageous for superior photocatalytic performance [40].

### 3.6. Photocatalytic Studies

**Scheme 2:**
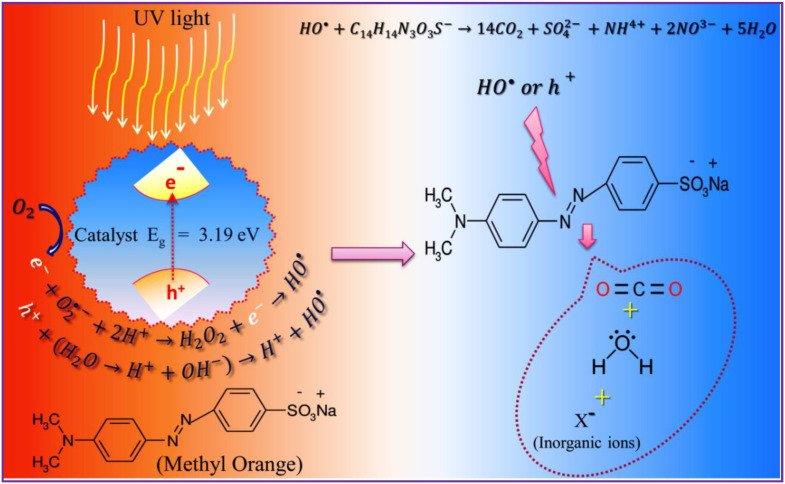
Schematic illustration of the photocatalytic mechanism of ZnO.SiO_2_ photocatalyst for degradation of MO.

Absorption of UV light with energy greater than the band gap of the catalyst material causes generation of electron-hole pairs in ZnO particles i.e., electrons (e^−^) in conduction band and holes (h^+^) in the valence band holes. The holes react with H_2_O or hydroxide ions, which are adsorbed on the surface of the ZnO particles and produce ^•^OH. Conversely, the electron reduces O_2_ and produces O_2_^•−^ as well as other oxygen species (H_2_O_2_ and ^•^OH) [32,33]. Both the holes and the ^•^OH are super reactive to organic compounds that are in contact with these radicals [33]. The oxidizing tendency of the ^•^OH radicals is high enough to split C-H as well as C-C bonds of MO sitting on the outer surface of ZnO.SiO_2_ nanocomposite leading to CO_2_ and H_2_O production as shown in Scheme 2.

**Figure 7:**
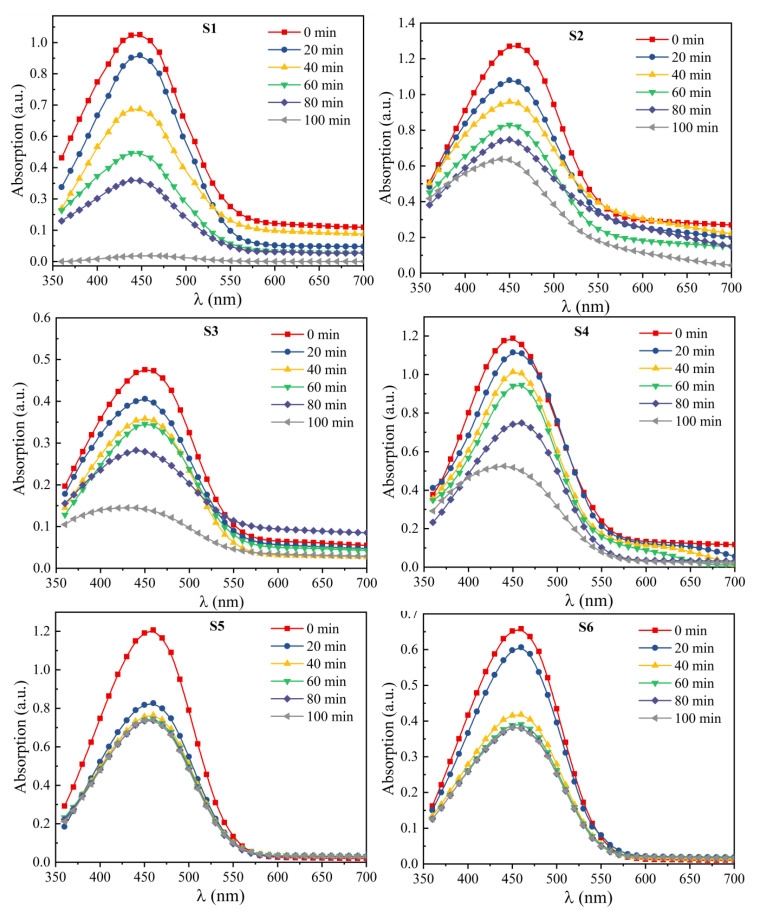
UV–visible spectra of S1, S2, S3, S4, S5, and S6 samples showing degradation of MO.

The ability of photocatalyst to decompose the pollutant compound MO, depends upon the different factors, such as the annealing temperature of the photocatalyst, the pH value of solution containing MO dye, and the time of irradiation and different conc. of NaOH. The effect of these factors on the catalytic performance of the material is examined. For an investigation of the effect of pH conc. and annealing temperatures of catalytic material on MO dye, samples of MO and ZS1 (annealed at 300, 600 & 900 °C) were prepared distinctly with different pH concentrations. The samples’ names are abbreviated as S1 (ZS1_300_, pH7), S2 (ZS1_300_, pH12), S3 (ZS1_600_, pH7), S4 (ZS1_600_, pH12), S5 (ZS1_900_, pH7), and S6 (ZS1_900_, pH12). The UV–visible spectra of each sample showing degradation of MO w.r.t. irradiation time, depicted in Figure 7.

#### 3.6.1. Effect of pH and Irradiation Time

**Figure 8:**
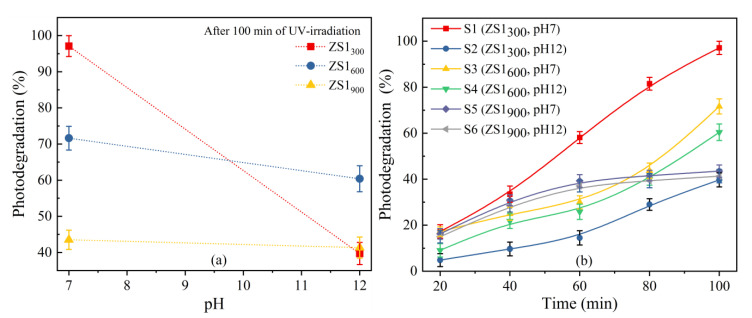
Effect of (**a**) irradiation time and (**b**) pH on photodegradation rate (%) of methyl orange (UV irradiation = (2 × 125) W).

Using Equation (1), the photo-degradation percentages of samples S1, S2, S3, S4, S5, and S6 irradiated under the UV light for 100 min are calculated and the values of photo-degradation (%) are found to be 98.1, 39.7, 71.63, 60.39, 43.51, and 41.34%, respectively (Figure 8a). Interestingly, it is observed that the photocatalytic activity of the S1 sample is relatively higher than the other samples. As the value of pH is increased, the degradation response is decreased and only 39.7 % of MO degradation response is observed for sample S2 (i.e., at pH = 12). The photo-degradation activity depends on the surface properties of the catalyst. Generally, pH changes the surface properties of catalysts like surface charge properties that oversee the absorption mechanism of MO on catalytic surface. At a pH 7 (below ZPC pH i.e., 8), the surface of ZnO is positively charged, which can absorb negatively charged MO due to electrostatic attraction, facilitating the degradation reaction. In contrast, at a higher pH (=12), absorption is decreased due to electrostatic repulsive forces among the catalytic surface and the dyed surface. As a result, photo-degradation response becomes minuscule; this is firstly because of the decrease in adsorption of the dye molecules on surface of the photocatalyst surface at higher pH [41,42], and secondly due to rapid scavenging of the hydroxyl radicals produced via reaction between holes and OH^−^, which causes a decrease in free radicals [42,43]. It is reported that pH affects the production of these free radicals that, in turn, lower the degradation rate of dye, or the termination of degradation [31].

It is observed that on increasing the irradiation time under light irradiating lamps, the photo-degradation percentage of methyl orange also increased, as shown in Figure 8b. It is observed from Figure 8b that all the samples attained maximum photo-degradation (%) in about 100 min. This may be due to the production of more and more e-h pairs and the corresponding excitation of an electron from the valence band to the conduction band [44]. Accordingly, the production of hydroxyl radicals is rose, which resulted in an enhanced photodegradation (%). The higher degradation efficiency of the ZS1_300_ nanocomposite sample, as compared to other samples, can be attributed to its small band gap and larger surface area.

#### 3.6.2. Effect of Annealing Temperatures

**Figure 9:**
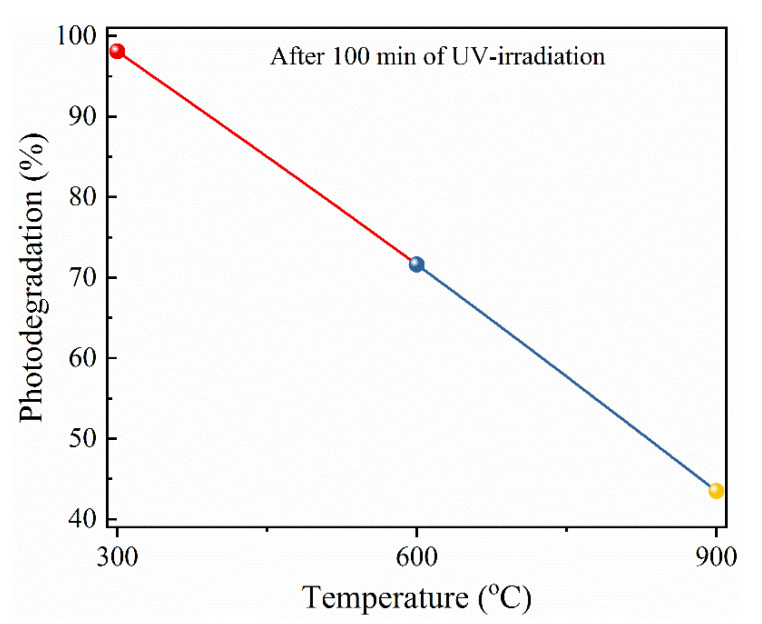
Variation in photo-degradation performance of ZS1 catalyst w.r.t. annealing temperature at pH 7 after 100 min of UV exposure.

The photocatalytic efficiency of the ZS1 nanocomposite at different annealing temperatures has also been assessed for the photo-degradation of MO. Figure 9 shows the correlation between photo-degradation (%) of MO with annealing temperatures of ZS1 nanocomposite. It is observed that the photocatalytic degradation of MO decreases from 98.1% to about 39% with an increase in the annealing temperature of the sample ZS1 (i.e., sample ZS1_300_ to ZS1_900_. The highest activity observed for the ZS1_300_ nanocomposite may be ascribed to the higher specific surface area, lower band gap value, and efficient charge separation ability of the sample through the charge exchange process in the existence of UV light. The lowest photo-degradation ability of the sample ZS1_900_ can be due to the decrease in specific surface area due to the filling of the pore by the penetration of ZnO into the SiO_2_ porous matrix, as observed from the XRD and BET studies. The lower reactivity of SiO_2_ towards photo-degradation and the presence of higher content of SiO_2_ on the upper surface as expected from XRD pattern could be another reason for the lower efficiency of ZS1_900_ sample.

It is reported that in the photocatalytic mechanism, the production of ^•^OH is expected to occur mainly on an internal surface i.e., the ZnO surface [45,46]. In addition, MO approaches the ZnO surface via the pores present in the surface area of sample ZS1. Thus, the photonic degradation efficiency is being limited by the absorption or diffusion rate of the MO molecule into the photo-catalytically active surface. As a result of the difference in the size and the number of pores of the ZS1 samples (ZS1_300_, ZS1_600_ & ZS1_900_,), the photo-degradation (%) is changed accordingly. Therefore, it is concluded that the ZS1_300_ assists the transport properties of all reactants involved in the photocatalytic process, which enhanced the overall photocatalytic activity.

#### 3.6.3. Effect of NaOH Concentration

**Figure 10:**
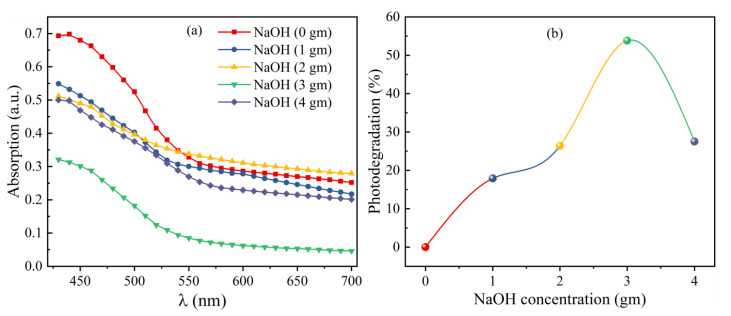
Effect of NaOH on catalyst; (**a**) UV–visible spectra for different concentrations of NaOH and (**b**) photo-degradation efficiency w.r.t. concentration NaOH.

To investigate the effect of NaOH concentration on the photo-degradation ability of the nanocomposite samples, four different concentrations (1, 2, 3 and 4 g) of NaOH are mixed with the photocatalyst and MO solution. In a typical process, 0.38 g of catalyst powder was dispersed in 100 mL deionized water and then 10 mL of methyl orange dye solution was added to catalyst solution and stirred to adsorb the dye molecules on surface of the photocatalyst. After that, 1 g of NaOH powder was mixed with the above solution containing methyl orange and photocatalyst, then the solution was stirred in absence of light to achieve equilibrium between adsorption and desorption and then irradiated with visible irradiation light with continuous stirring [47]. After the decoloration of methyl orange, a part of the solution was used for recording the absorbance spectrum using a UV-Visible spectrophotometer and photo-degradation percentage was calculated using Equation (1). The same steps are followed for the degradation of MO with different weights (2, 3 and 4 g) of NaOH and the recorded absorption spectra are shown in Figure 10a.

It is observed that the decoloration time of methyl orange decreases from 275 through 95 to 40 min with an increase in NaOH concentration from 1 g through 2 g to 3 g. However, for the 4 g of NaOH, again the decoloration time increases to 80 min. It is observed that the sample containing 3 g concentration of NaOH shows a higher photo-degradation of 53.8 %, and beyond 3 g NaOH concentration again the photo-degradation (%) decreases to 27.5%, with 4 g of NaOH (Figure 10b). The results showed that the photo-degradation rate increased with an increase in NaOH concentration up to 3 g of NaOH, which may be due to OH^−^ ions produced by NaOH acting as a strong hole scavenger and generating hydroxyl radicals, thus reducing the e-h pair recombination [45]. Moreover, with a further increase in concentration (>3 g) of NaOH, the degradation rate became poor, since a greater amount of hydroxyl radicals may oxidize the catalyst [45].

Compared to other metal oxide composites tabulated in Table 2, the present ZnO.SiO_2_ prepared through the co-precipitation method shows enhanced photocatalytic efficiency for the degradation of MO.

## 4. Conclusions

ZnO.SiO_2_ nanocomposites are successfully synthesized via the co-precipitation method. The specific surface area decreases from 16.8 m^2^/g and 0.001 m^2^/g on increasing the annealing temperature from 300 to 900 °C due to blocking of SiO_2_ pores via the penetration of ZnO into these pores. It is found that the specific surface area of the nanocomposite plays an important role in photocatalytic activities and the degradation of methyl orange dye. The effect of various parameters, such as the pH value of the solution, irradiation time, annealing temperature, and NaOH concentration on photocatalytic performance of ZS1 nanocomposite have been investigated. The sample ZS1_300_ shows a higher photo-degradation efficiency of 98.1% at pH 7 for 100 min irradiation times. The high efficiency of ZS1_300_ can be governed by the following mechanisms: (i) a higher specific area that assures the maximum interaction with UV irradiation; (ii) an optimized bandgap resulting in enhanced absorption efficiency of UV irradiation where more electron-hole pairs are produced; (iii) more irradiation time proportional to more generation electron-hole pairs; and (iv) at lower pH below the ZPC pH, which increases the absorption of pollutant dye that resulting higher efficiency. OH^−^ radicals act as active species for the degradation of the pollutant. For the degradation, the formation of hole and hydroxyl radicals is proven to be the crucial factor. The results revealed that ZnO.SiO_2_ nanocomposite is an effective, inexpensive, and eco-friendly catalyst for the degradation of methyl organic pollutants.

## Figures and Tables

**Table 1:** Average crystallite size (*D*), bandgap energy (*E_g_*) and grain boundary specific area (*S_a_*) w.r.t. to annealing temperatures.

Annealing Temp (°C)	*D* (nm)	*S_a_* (m^2^·m^−3^)	*E_g_* (eV)
300	36.20	4.455 × 10^7^	3.26
600	38.81	4.251 × 10^7^	3.25
900	43.42	3.800 × 10^7^	3.36

**Table 2:** Photocatalytic activity of various ZnO composites.

Catalyst Composite	Method of Synthesis	Light Used for Irradiation	Irradiation Time (min)	Photo-Degradation (%)	Refs.
ZnO nano-needles on Si substrates	Hydrothermal	UV light	140	95.4	[48]
ZnO quantum dots	Flame spray pyrolysis	UV light	160	97	[49]
TiO_2_/biochar	Hydrolysis	UV light	160	96.88	[17]
Xanthan gum/TiO_2_	Hydro-gel	Visible light	90	89	[15]
Anatase TiO_2_	Sol-gel	Visible light	240	67.12	[50]
ZnO catalyst	Hydrothermal	UV light	180	99.7	[51]
Rare earth metal doped ZnO	Sol-gel	UV light	180	98	[32]
ZnO.SiO_2_	Co-precipitation	UV light	100	98.1	Present work

## Supplementary Materials

The following are available online at www.mdpi.com/article/10.3390/nano11102548/s1, Table S1: Structural parameters of ZS1300 achieved from X-ray Diffraction Pattern, Table S2: Fractional atomic coordinates and isothermal parameter of different atoms obtained from the Rietveld anal-ysis of XRD patterns for the sample ZS1300, Table S3: Bond Length and bond angles of sample ZS1300, Table S4: Structural parameters of ZS1600 achieved from X-ray Diffraction Pattern, Table S5: Structural parameters of ZS1900 achieved from X-ray Diffraction Pattern, Table S6: Structural parameters of ZS2300 achieved from X-ray Diffraction, Table S7: Structural parameters of ZS2600 achieved from X-ray Diffraction Pattern.
