# Supplementary material for: Excellent UV-Light Triggered Photocatalytic Performance of ZnO.SiO2 Nanocomposite for Water Pollutant Compound Methyl Orange Dye
Source: Nanomaterials (Basel). 2021 Sep 28;11(10):2548. doi: 10.3390/nano11102548 (PMC8541141; doi:10.3390/nano11102548)
Supplement: Supplementary file 1 [file nanomaterials-11-02548-s001.zip › nanomaterials-1390121-supplementary.pdf]

nanomaterials

Supplementary Materials

Excellent UV-Light Triggered Photocatalytic Performance of $ZnO.SiO_2$ Nanocomposite for Water Pollutant Compound Methyl Orange Dye

Sunil Rohilla [1], Ankita Gupta [2], Vibhor Kumar [3], Suman Kumari [1], Michal Petru [4], Nesrine Amor [4], Muhammad Tayyab Noman [4] and Jasvir Dalal [1,*]

1 Department of Physics, Chaudhary Ranbir Singh University, Jind 126102, India; sunilrohilla@crsu.ac.in (S.R.); sumankkharb@gmail.com (S.K.)
2 Department of Physics, Adarsh Mahila Mahavidyalaya, Bhiwani 127021, India; ankita13994@gmail.com
3 Department of Electrical Engineering, Northern Illinois University, Dekalb, IL 60115, USA; vibhorsoni123@gmail.com
4 Department of Machinery Construction, Institute for Nanomaterials, Advanced Technologies and Innovation, Technical University of Liberec, Studentská 46117, Czech Republic; michal.petru@tul.cz (M.P.); nesrine.amor@tul.cz (N.A.); tayyab_noman411@yahoo.com (M.T.N.)
* Correspondence: tayyab_noman411@yahoo.com (M.T.N.); jasvirdalal2012@gmail.com (J.D.)

Table S1. Structural parameters of $ZS1_{300}$ achieved from X-ray Diffraction Pattern.

S. no.	2-theta	d-spacing	Intensity	(hkl)	FWHM	Crystallite Size(nm)	Lattice Strain	Dislocation Density (10^{-4} nm^{-2})	Microstrain
1	31.851	2.809	557	(100)	0.2400	35.97	0.0037	7.7	0.0576
2	34.552	2.599	410	(002)	0.2400	36.22	0.0034	7.6	0.0572
3	36.358	2.471	999	(101)	0.2400	36.41	0.0032	7.5	0.0570
4	47.698	0.908	218	(102)	0.2400	37.82	0.0024	6.9	0.0548
5	56.751	1.625	318	(110)	0.2800	33.69	0.0023	8.8	0.0615
6	63.093	1.485	284	(103)	0.3200	30.43	0.0023	10.8	0.0681
7	66.565	1.406	43	(200)	0.2000	49.64	0.0013	4.0	0.0417
8	68.168	1.378	235	(112)	0.2000	50.11	0.0013	3.9	0.0414
9	69.291	1.357	116	(201)	0.2800	36.03	0.0018	7.7	0.0575

Table S2. Fractional atomic coordinates and isothermal parameter of different atoms obtained from the Rietveld analysis of XRD patterns for the sample $ZS1_{300}$.

Parameters→ Atoms ↓	x/a	y/b	z/c	Wyckoff positions
Zn	1/3	2/3	½	2b
O	2/3	1/3	3/8	2b
Si1	0.255(8)	0.255(8)	0.255(8)	4a
Si2	−0.008(8)	−0.008(8)	−0.008(8)	4a
O1	0.125(15)	0.125(15)	0.125(15)	4a
O2	0.660(15)	0.660(15)	0.062(15)	12b

Table S3. Bond Length and bond angles of sample $ZS1_{300}$.

S. no.	Atom 1	Atom 2	d 1,2[Å]	A	d 1,3[Å]	Angle 2,1,3[Å]
1	Zn	O	1.973	O	1.973	0.094
2	Zn	O	1.973	O	1.973	0.094
3	Zn	O	1.973	O	1.977	108.579
4	Zn	O	1.973	O	1.977	108.579
5	Zn	O	1.973	O	1.977	108.579
6	Zn	O	1.973	O	1.977	108.579
7	Zn	O	1.973	O	1.9797	108.553
8	Zn	O	1.973	O	1.9797	108.553
9	Zn	O	1.973	O	1.9797	108.553
10	Zn	O	1.973	O	1.9797	108.553
11	Zn	O	1.973	O	1.9824	108.527
12	Zn	O	1.973	Zn	3.2073	35.753
13	Zn	O	1.973	Zn	3.2073	35.753
14	Zn	O	1.973	Zn	3.2073	144.247
15	Zn	O	1.973	Zn	3.2073	144.247
16	Zn	O	1.973	Zn	3.2073	35.753
17	Zn	O	1.973	Zn	3.2073	35.753
18	Zn	O	1.973	Zn	3.2073	144.247
19	Zn	O	1.973	Zn	3.2073	144.247
20	Zn	O	1.973	Zn	3.209	144.207
21	Zn	O	1.973	Zn	3.209	144.207
22	Zn	O	1.973	Zn	3.209	35.793
23	Zn	O	1.973	Zn	3.209	35.793
24	Zn	O	1.973	Zn	3.209	35.793
25	Zn	O	1.973	Zn	3.209	144.207
26	Zn	O	1.973	Zn	3.209	144.207
27	Zn	O	1.973	Zn	3.209	35.793
28	Zn	O	1.973	Zn	3.2106	144.166
29	Zn	O	1.973	Zn	3.2106	35.834
30	Zn	O	1.973	O	3.2328	180
31	Zn	O	1.973	O	3.2328	179.942
32	Zn	O	1.973	O	3.2328	179.942
33	Zn	O	1.973	Zn	3.2459	90
34	Zn	O	1.973	Zn	3.2459	90
35	Zn	O	1.973	Zn	3.2475	90
36	Zn	O	1.973	Zn	3.2475	90
37	Zn	O	1.973	Zn	3.2475	90
38	Zn	O	1.973	Zn	3.2475	90
39	Zn	O	1.973	Zn	3.2491	90
40	Zn	O	1.973	Zn	3.2491	90
41	Zn	O	1.973	Zn	3.2491	90
42	Zn	O	1.973	Zn	3.2491	90
43	Zn	O	1.973	Zn	3.2491	90
44	Zn	O	1.973	Zn	3.2491	90
45	Zn	O	1.973	Zn	3.2507	90
46	Zn	O	1.973	Zn	3.2507	90
47	Zn	O	1.973	Zn	3.2507	90

48	Zn	O	1.973	Zn	3.2507	90
49	Zn	O	1.973	Zn	3.2523	90
50	Zn	O	1.973	Zn	3.2523	90
51	Zn	O	1.973	O	1.973	0.094
52	Zn	O	1.973	O	1.977	108.661
53	Zn	O	1.973	O	1.977	108.579
54	Zn	O	1.973	O	1.977	108.579
55	Zn	O	1.973	O	1.977	108.661
56	Zn	O	1.973	O	1.9797	108.553
57	Zn	O	1.973	O	1.9797	108.635
58	Zn	O	1.973	O	1.9797	108.471
59	Zn	O	1.973	O	1.9797	108.471
60	Zn	O	1.973	O	1.9824	108.445
61	Zn	O	1.973	Zn	3.2073	35.834
62	Zn	O	1.973	Zn	3.2073	35.753
63	Zn	O	1.973	Zn	3.2073	144.329
64	Zn	O	1.973	Zn	3.2073	144.247
65	Zn	O	1.973	Zn	3.2073	35.752
66	Zn	O	1.973	Zn	3.2073	35.834
67	Zn	O	1.973	Zn	3.2073	144.329
68	Zn	O	1.973	Zn	3.2073	144.247
69	Zn	O	1.973	Zn	3.209	144.207
70	Zn	o	1.973	Zn	3.209	144.125
71	Zn	o	1.973	Zn	3.209	35.793
72	Zn	o	1.973	Zn	3.209	35.712
73	Zn	o	1.973	Zn	3.209	35.712
74	Zn	o	1.973	Zn	3.209	144.288
75	Zn	o	1.973	Zn	3.209	144.125
76	Zn	o	1.973	Zn	3.209	35.875
77	Zn	o	1.973	Zn	3.2106	144.084
78	Zn	o	1.973	Zn	3.2106	35.752
79	Zn	O	1.973	O	3.2328	179.906
80	Zn	O	1.973	O	3.2328	179.867
81	Zn	O	1.973	O	3.2328	179.848
82	Zn	O	1.973	Zn	3.2459	90.094
83	Zn	O	1.973	Zn	3.2459	90.047

Table S4. Structural parameters of $ZS1_{600}$ achieved from X-ray Diffraction Pattern.

Scheme 2	2-theta	Intensity	FWHM	Crystallite Size (nm)	Dislocation Density (nm^{-2})	Microstrain
1	31.78	6147	0.21926	39.36	0.00064549	0.05272046
2	34.44	4364	0.22729	38.23	0.00068421	0.05427543
3	36.279	9574	0.2249	38.84	0.00066289	0.05343069
4	47.538	2134	0.27084	33.49	0.000892	0.061966
5	56.597	2973	0.29154	32.34	0.000956	0.064174
6	62.837	2495	0.32077	30.33	0.001087	0.068435
7	66.376	633	0.37468	26.48	0.001426	0.07839
8	67.956	2072	0.34616	28.92	0.001196	0.071764
9	69.096	1133	0.35454	28.43	0.001237	0.073004

Table S5. Structural parameters of $ZS1_{900}$ achieved from X-ray Diffraction Pattern.

Sr. no.	2-theta	Intensity	FWHM	Crystallite Size (nm)	Dislocation Density (nm^{-2})	Microstrain
1	13.05325	751.9	0.23867	35.01	0.00081586	0.05928079
2	22.17324	9018.9	0.190000	44.52	0.000504533	0.04661318
3	24.49324	790.4	0.40496	20.97	0.002274066	0.09893614
4	24.97324	1120.9	0.3321	25.6	0.001525879	0.08106113
5	25.89324	4269.9	0.200000	42.59	0.000551296	0.04872895
6	28.07324	866.4	0.72996	11.72	0.007280225	0.17704101
7	28.67324	855.4	0.55142	15.54	0.004140931	0.13356178
8	29.53324	597.4	0.65764	13.05	0.005871904	0.15897987
9	31.89323	4009.9	0.20000	43.16	0.00053683	0.0480759
10	34.37323	3838.9	0.21682	40.07	0.000622818	0.05178458
11	36.35323	1900.9	0.938	9.31	0.011537206	0.22279822
12	39.21323	1735.9	0.31222	28.22	0.0012557	0.07352926
13	41.19323	533.4	0.90165	9.84	0.010327847	0.2110042
14	43.09322	569.9	1.12895	7.91	0.015982585	0.26251449
15	45.39322	651.9	1.31756	6.83	0.021436733	0.30388233
16	47.31321	705.4	0.88918	10.19	0.009630562	0.20361466
17	49.29321	1451.9	0.39168	23.32	0.001838836	0.08899921
18	54.6332	461.9	1.31285	7.12	0.019726045	0.29161165
19	56.5532	630.4	1.55808	6.05	0.027320538	0.34303893
20	58.0132	637.9	0.68883	13.78	0.005266251	0.15060646
21	59.99319	568.9	1.09485	8.76	0.013031421	0.2370501
22	61.29319	622.4	0.8292	11.64	0.00738064	0.17834594
23	66.05318	1296.9	0.61068	16.21	0.003805695	0.12800123
24	69.03318	685.9	0.93018	10.83	0.008525956	0.19160828

Table S6. Structural parameters of $ZS2_{300}$ achieved from X-ray Diffraction.

Sr. no.	2-theta	Intensity	FWHM	Crystallite Size (nm)	Dislocation Density (nm^{-2})	Microstrain
1	31.78	7344.031	0.25029	34.48	0.000841	0.060181
2	34.42	6553.031	0.25686	33.83	0.000874	0.061339
3	36.23	10116.03	0.26699	32.71	0.000935	0.063439
4	47.558	4609.031	0.28763	31.54	0.001005	0.065803
5	56.597	5554.031	0.35057	26.89	0.001383	0.07716
6	62.877	5106.031	0.3907	24.9	0.001613	0.083336
7	66.3766	3578.731	0.39489	25.12	0.001585	0.082611
8	67.976	5027.031	0.37244	26.88	0.001384	0.077202
9	69.076	4055.031	0.40457	24.91	0.001612	0.083316

Table S7. Structural parameters of $ZS2_{600}$ achieved from X-ray Diffraction Pattern.

Sr. no.	2-theta	Intensity	FWHM	Crystallite Size (nm)	Dislocation Density (nm^{-2})	Microstrain
1	31.78022	5888.5	0.25	34.52	0.000839	0.060112
2	34.44003	4288.5	0.24909	34.89	0.000821	0.059481
3	36.27989	9721.5	0.25356	34.45	0.000843	0.060239
4	47.55886	2053.5	0.28763	31.54	0.001005	0.065803
5	56.59786	2998.5	0.30626	30.78	0.001056	0.067414
6	62.87707	2550.5	0.32788	29.68	0.001135	0.069937
7	66.3766	423.2	0.39489	25.12	0.001585	0.082619
8	67.95638	2124.5	0.3447	29.04	0.001186	0.071461
9	69.07622	1049.5	0.35373	28.49	0.001232	0.072846
